# Evaluation and comparison of the potential of two ferritins as anti-tick vaccines against *Haemaphysalis longicornis*

**DOI:** 10.1186/s13071-014-0482-x

**Published:** 2014-10-12

**Authors:** Remil Linggatong Galay, Takeshi Miyata, Rika Umemiya-Shirafuji, Hiroki Maeda, Kodai Kusakisako, Naotoshi Tsuji, Masami Mochizuki, Kozo Fujisaki, Tetsuya Tanaka

**Affiliations:** Department of Pathological and Preventive Veterinary Science, The United Graduate School of Veterinary Science, Yamaguchi University, Yoshida, Yamaguchi 753-8515 Japan; Laboratory of Infectious Diseases, Joint Faculty of Veterinary Medicine, Kagoshima University, 1-21-24 Korimoto, Kagoshima, 890-0065 Japan; Laboratory of Food Chemistry, Department of Biochemistry and Biotechnology, Division of Molecular Functions of Food, Faculty of Agriculture, Kagoshima University, 1-21-24 Korimoto, Kagoshima, 890-0065 Japan; National Research Center for Protozoan Diseases, Obihiro University of Agriculture and Veterinary Medicine, Inada-cho, Obihiro, Hokkaido 080-8555 Japan; Department of Parasitology, Kitasato University School of Medicine, Kanagawa, 252-0374 Japan; National Agricultural and Food Research Organization, 3-1-5 Kannondai, Tsukuba, Ibaraki 305-0856 Japan

**Keywords:** Ticks, *Haemaphysalis longicornis*, Ferritin, Vaccine, Iron metabolism, Oxidative stress

## Abstract

**Background:**

Tick control is an essential aspect of controlling the spread of tick-borne diseases affecting humans and animals, but it presently faces several challenges. Development of an anti-tick vaccine is aimed at designing cost-effective and environmentally friendly protection against ticks and tick-borne diseases as an alternative to the use of chemical acaricides. A single vaccine from the tick midgut protein Bm86 is currently available for field applications, but its efficacy is limited to only some tick species. Identification of candidate vaccine antigens that can affect multiple tick species is highly desirable. The hard tick *Haemaphysalis longicornis* has two kinds of the iron-binding protein ferritin (HlFER), an intracellular HlFER1 and a secretory HlFER2, and RNA interference experiments showed that these are physiologically important in blood feeding and reproduction and in protection against oxidative stress. Here we investigated the potential of targeting HlFERs for tick control by immunizing the host with recombinant HlFERs (rHlFER1 and rHlFER2).

**Methods:**

Rabbits were immunized with rHlFERs three times subcutaneously at two-week intervals. Antisera were collected before the first immunization and a week after each immunization to confirm the antigen-specific serum antibody titer by serum ELISA. Two weeks after the final immunization, the rabbits were challenged with tick infestation. After dropping, tick feeding and reproduction parameters were evaluated to determine vaccine efficacy. To demonstrate the effects of antibodies, oxidative stress was detected in the eggs and larvae.

**Results:**

The antibody titer of rHlFER-immunized rabbits greatly increased after the second immunization. Antibodies exhibited cross-reactivity with rHlFERs and reacted with tick native HlFERs in Western blot analysis. Significantly lower bodyweight was observed in the ticks infested from the rHlFER2-immunized rabbit compared to those from the control rabbit. Reduced oviposition and hatching rate were observed in both rHlFER-immunized groups. rHlFER2 showed a higher vaccine efficacy. The antibodies against rHlFERs were detected in the eggs, and higher levels of oxidative stress biomarkers in the eggs and larvae, of ticks from rHlFER vaccinated rabbits.

**Conclusion:**

Collectively, these results showed that HlFER2 has a good potential as an anti-tick vaccine antigen that may affect multiple tick species.

**Electronic supplementary material:**

The online version of this article (doi:10.1186/s13071-014-0482-x) contains supplementary material, which is available to authorized users.

## Background

Ticks and tick-borne diseases remain threats to human and animal health worldwide. Aside from the direct damage that ticks inflict on their host, they serve as vectors of a wide variety of pathogens, including protozoa, rickettsiae and viruses. In humans, Lyme borreliosis and tick-borne encephalitis are among the most important diseases transmitted by ticks [1]. In cattle, ticks are responsible for the spread and persistence of theileriosis, anaplasmosis, cowdriosis, and babesiosis [2]. The hard tick *Haemaphysalis longicornis,* mainly distributed in East Asia and Australia, is a known vector of babesiosis caused by *Babesia ovata, B. major*, *B. gibsoni*, and possibly *B. bigemina* and of theileriosis caused by *Theileria sergenti*, *T. orientalis*, and *T. buffeli* [3,4]. Recently, *H. longicornis* has been strongly implicated as a vector of severe fever with thrombocytopenia syndrome (SFTS) virus affecting humans, which has been reported in China [5], Japan [6] and South Korea [7].

Effective tick control is essential in preventing tick infestation and, subsequently, the spread of tick-borne pathogens. Until now, the use of chemical acaricides was the primary measure of controlling ticks worldwide. However, concerns about limited efficacy, the emergence of resistant ticks, and contamination of the environment and animal products are among the disadvantages of acaricide application. Vaccination is a promising control alternative that will avoid the drawbacks of acaricide application [8]. Ideally, these vaccines should reduce tick infestation and pathogen transmission [9]. For about 20 years, the only commercially available anti-tick vaccine has utilized the midgut protein Bm86 from *Rhipicephalus* (*Boophilus*) *microplus* as the antigen [10]; however, it is only effective against a limited number of tick species [8]. Although numerous antigens have been studied as candidates for a tick vaccine, no other tick vaccine has progressed to commercial development [11]. The main challenge in anti-tick vaccine development is the identification of a suitable tick protective antigen that can be effective against all developmental stages and a wide range of tick species. Many studies on ticks are now focused on the identification of antigens using combined approaches [12] aimed at targeting multiple tick species and multiple tick-borne pathogens at the same time [1,13,14].

Ferritin (FER) is generally an iron-binding protein consisting of 24 subunits folded in a helical bundle involved in iron homeostasis in most organisms [15]. Two types of ferritin, an intracellular (FER1) and a secretory type (FER2), have been characterized in the hard ticks *Ixodes ricinus* [16] and *H. longicornis* [17]. These molecules were found to be crucial in the blood feeding and reproduction of these hard ticks. Knockdown experiments through RNA interference (RNAi) in both studies resulted in reduced blood feeding capacity, high mortality after blood feeding, and reduced fecundity [16,17] as consequences of iron overload and oxidative stress [18]. These results implied that FERs of the hard ticks may be good target molecules for tick control. Hajdusek *et al.* [19] performed vaccination studies using recombinant FER2 against *I. ricinus* and *Rhipicephalus microplus*. Here we compared the potential of two recombinant FERs of *H. longicornis*, rHlFER1 and rHlFER2, as vaccines for tick control. We also attempted to demonstrate how vaccination using these rHlFERs can affect ticks by examining whether induced antibodies can block the function of native HlFERs.

## Methods

### Ticks and animals

Adult parthenogenetic (Okayama strain) *H. longicornis* ticks were used for the infestation challenge following host immunization. These ticks were maintained by feeding on the ears of Japanese white rabbits (Kyudo, Kumamoto, Japan) for several generations at the Laboratory of Infectious Diseases, Joint Faculty of Veterinary Medicine, Kagoshima University, Kagoshima, Japan [20]. Rabbits were also used for the whole immunization experiment. The animals were maintained and the experiments performed according to the approved guidelines from Animal Care and Use Committee of Kagoshima University (approval number VM13007).

### Expression and purification of recombinant ferritins

The open reading frame (ORF) of *Hlfer1* (GenBank: AY277905) or *Hlfer2* (GenBank: AB734098) was extracted from their respective pGCAP1 vector using the following primer sets with *Bam*HI recognition sites: Hlfer1-F 5′-ACGGATCCAAAATGGCCGCTACT-3′ and Hlfer1-R 5′-ACGGATCCTCCTCAGTCGTCTCC-3′ for rHlFER1 or Hlfer2-F 5′-ACGGATCCACCATGCTCCCGATC-3′ and Hlfer2-R 5′-ACGGATCCGGTTTATTTGTCGCT-3′ for rHlFER2. After cutting with *Bam*HI, the amplified DNA fragments were purified using GENECLEAN II kit (MP Biomedicals LLC, Solon, OH, USA) and then subcloned into the *Bam*HI cutting site of pRSET A vector (Invitrogen, Carlsbad, CA, USA). The constructs, pRSETA/HlFER1 and pRSETA/HlFER2, were expressed in *E. coli* BL21 (DE3) cells grown in Luria-Bertani broth medium with ampicillin. The expression of histidine (His)-tagged rHlFERs was induced with 1 mM final concentration of isopropyl β-D-1-thiogalactopyranoside (IPTG). After overnight induction, cells were collected by centrifugation at 3,350×*g* for 30 min, and the proteins were extracted through ultrasonication. Purification was done through affinity chromatography using a His-trap™ FF column (GE Healthcare, Uppsala, Sweden) at denatured condition with 6 M urea, followed by dialysis, first against phosphate-buffered saline (PBS) containing 0.5 M arginine for refolding overnight, and then against PBS alone overnight. The purity of the rHlFERs was confirmed by SDS-PAGE, and the concentration was determined through SDS-PAGE using bovine serum albumin as the standard and Micro BCA Assay kit (Thermo Scientific, Rockford, IL, USA). The rHlFERs were kept at −30°C until use.

### Rabbit immunization

A total of three rabbits from each group were used for two separate vaccination trials. For each immunization, rHlFER1 or rHlFER2 was thoroughly mixed with an equal volume of incomplete Freund’s adjuvant (Sigma-Aldrich, St. Louis, MO, USA) to a final concentration of 100 μg per 1.5 mL. The mixture was administered subcutaneously using a sterilized glass syringe and a 21G needle and repeated three times at two-week intervals. Control rabbits were immunized with adjuvant alone. Sera were collected before the first immunization and a week after each immunization for confirmation of antibody titer (days 0, 7, 21, and 35).

### Measurement of serum antibody levels through ELISA

The antigen-specific serum antibody titer was determined by ELISA. ELISA plates (F96 Maxisorp, Nunc, Roskide, Denmark) were coated with either rHlFER1 or rHlFER2 dissolved in a carbonate buffer (pH 9.6) at 100 ng/100 μl per well concentration at 4°C overnight. Another recombinant protein prepared in our laboratory with His-tag, recombinant peroxiredoxin2 of *H. longicornis* (HlPrx2), was used as a control antigen (Kusakisako *et al.,* unpublished results). After washing with PBS with 0.05% Tween 20 (PBS-T), each well was blocked with 150 μl of 5% skimmed milk in PBS-T at 37°C for 1 h. The plates were incubated with 100 μl/well of rabbit sera in the blocking solution, diluted serially starting at 1:50, at 37°C for 1 h. ELISA plates were washed several times with PBS-T before applying 100 μl of HRP-conjugated polyclonal goat anti-rabbit immunoglobulins (Dako Cytomation, Glostrup, Denmark) in the blocking solution (1:2,000 dilution) in each well and then incubated at 37°C for 1 h. After another series of washing, 100 μl of TMB One Component HRP Microwell substrate (SurModics, Inc., Eden Prairie, MN, USA) was placed in each well and then incubated at 37°C for 30 min. The reaction was stopped by adding 100 μl of a mixture of 0.6 N H_2_SO_4_ and 1 N HCl (1:1) in each well. Absorbance was measured using a microplate reader (Bio-Rad, Hercules, CA, USA) at OD_450_.

### Tick infestation and evaluation of vaccination efficacy

Two weeks after the final immunization, 30 adult female ticks were infested on the ears of each rabbit as described previously [18] until they fed to repletion. After dropping, ticks were weighed and then monitored for survival rate, egg laying and subsequent hatching to larvae as previously described [17]. The effect of immunization was evaluated based on the reduction of the tick’s engorged body weight, oviposition, and hatching. Calculations were made using formulas adapted from previous tick vaccination reports [19].

Reduction of tick engorged weight (R_W_) = 100[1 – (BWV/BWC)], where BWV is the average engorged weight of ticks infested on rHlFER vaccinated rabbits and BWC is the average engorged weight of ticks infested on the control rabbits.

Reduction of oviposition (R_O_) = 100[1 – (EWV/EWC)], where EWV is the average weight of the eggs from ticks infested on rHlFER vaccinated rabbits and EWC is the average weight of the eggs from ticks infested on the control rabbits.

Reduction on hatching (R_H_) = 100[1 – (AHV/AHC)], where AHV is the percent of ticks with completely hatched eggs from the total number of ticks that laid eggs from rHlFER vaccinated rabbits and AHC is the percent of ticks with completely hatched eggs to the total number of ticks that laid eggs from the control rabbits.

Finally, the overall vaccine efficacy (E) for each group was calculated as 100[1 – (E_W_ × E_O_ × E_H_)], where E_W_ = BWV/BWC, E_O_ = EWV/EWC, and E_H_ = AHV/AHC.

### Tick protein preparation and Western blot analyses

Tick protein samples were prepared from unfed whole adults, partially fed midguts, eggs (20 days after laying), and newly hatched larvae by homogenizing them and suspending in PBS. After sonication, the tick homogenates were centrifuged and the supernatants were collected. The protein concentration of eggs and larvae was determined using Micro BCA kit (Thermo Scientific). To confirm the reactivity of rabbit sera to native tick HlFERs, whole adult and midgut protein samples were separated through SDS-PAGE and then transferred to a polyvinylidene difluoride membrane (Millipore, Bedford, MA, USA). After blocking with 5% skimmed milk in PBS-T overnight, the membrane was incubated with rabbit sera collected after the third immunization (1:100 dilution) as primary antibodies. Western blot analysis was also performed to further demonstrate the reactivity of rabbit antibodies to rHlFER1, rHlFER2, and rHlPrx2 as a control, with the primary antibodies diluted to 1:3,000. To detect the presence of antibodies in the eggs, recombinant proteins were used as protein samples, and then egg homogenates were used as primary antibodies (20 μg/500 μL). To demonstrate oxidative stress in eggs and larvae, malondialdehyde (MDA) and protein carbonyl were detected using specific kits for these oxidative stress markers (OxiSelect, Cell Biolabs, Inc., San Diego, CA, USA) as described previously [18]. Tubulin was detected as an internal control using a mouse-derived serum [21]. After incubation with HRP-conjugated goat anti-rabbit or anti-mouse immunoglobulin as a secondary antibody (1:30,000 dilution; Dako Cytomation), protein signals were detected using the ECL Prime Western Blotting Detection Reagent (GE Healthcare, Little Chalfont, Buckinghamshire, UK) and images were taken using the FluorChem FC2 Imaging System (Protein Simple, Santa Clara, CA, USA).

### Statistical analysis

The average values of two vaccination trials were calculated and statistical significance was determined using the Student’s *t*-test, with significant difference defined by *P* <0.05.

## Results

### Purification of recombinant HlFERs and rabbit immunization

**Figure 1:**
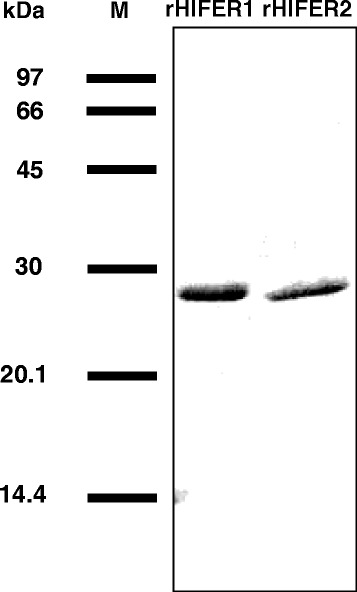
**Purification of recombinant HlFERs (rHlFERs).** His-tagged rHlFERs (rHlFER1 and rHlFER2) were expressed in *E. coli* and then purified through Ni-affinity chromatography and dialysis against PBS. After refolding, 2 μg per protein was subjected to SDS-PAGE, and then the gel was stained with Coomassie brilliant blue. M, low molecular weight marker.

**Figure 2:**
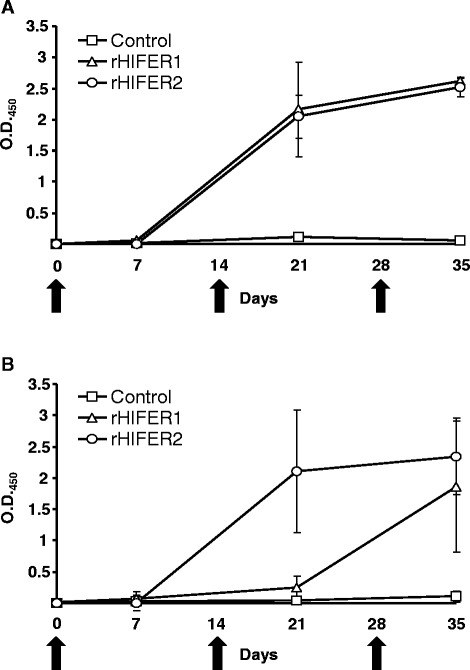
**Antigen-specific antibody titer against recombinant HlFER1 (rHlFER1) (A) and recombinant HlFER2 (rHlFER2) (B).** Rabbit sera were collected for serum ELISA at days 0, 7, 21, and 35. Antibody titers reflect the mean absorbance at OD_450_ of sera (10^4^ dilution) for each vaccinated group from two separate trials (n = 3). The times of immunizations are indicated by arrows. Control, rabbits injected with adjuvant only; rHlFER1, rabbits injected with rHlFER1; rHlFER2, rabbits injected with rHlFER2. Bars represent standard deviation.

rHlFERs were expressed and purified from *E. coli* cells. SDS-PAGE showed that the purified rHlFER1 and rHlFER2 have a similar molecular mass of around 26 kDa (Figure 1) as described previously [17]. An antigen-specific ELISA was conducted to monitor the antibody titer of individual rabbits, and then the average antibody titer for each group from the two trials was calculated (Figure 2). Whereas no changes were observed in the antibody titers of the control group, the antibody titers of the groups immunized against rHlFER1 (Figure 2A) and rHlFER2 (Figure 2B) significantly increased after the second immunization. Furthermore, the antibodies also exhibited cross-reactivity to each antigen. rHlFER1-immunized rabbits showed an abrupt increase in antibody titer against rHlFER1 after the second immunization, which further increased after the third immunization, while the titer for rHlFER2 only significantly increased after the third immunization. For rHlFER2-immunized rabbits, the antibody titer against rHlFER2 abruptly increased after the second immunization but did not significantly increase further after the third immunization, while the level and the trend of the antibody titer against rHlFER1 were similar to those of the rHlFER1-immunized rabbits. Cross-reactivity of the antibodies was also observed on ELISA using rHlPrx2 as antigen but it was lower than that observed in HlFERs (Additional file 1: Figure S1).

### Reactivity of rabbit antibodies to recombinant and native tick HlFERs

**Figure 3:**
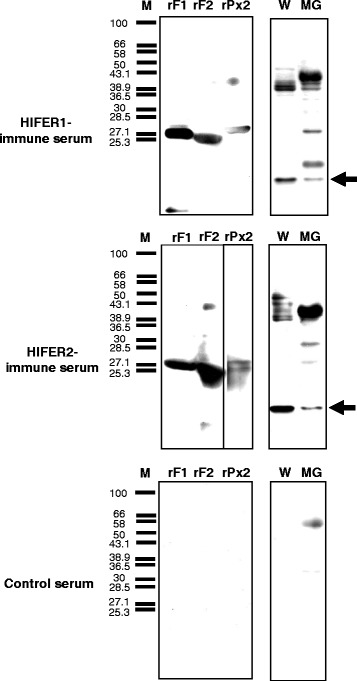
**HlFER detection using immunized rabbit sera.** Western blot analysis was performed to examine the reactivity of antibodies from vaccinated rabbits against recombinant HlFER1 (rF1), recombinant HlFER2 (rF2), recombinant HlPrx2 (rPx2) and native HlFERs of unfed whole ticks (W) and partially-fed midguts (MG). M, low molecular weight marker. Arrows point to positive bands for native tick HlFER1 or HlFER2 with around 20 kDa molecular weight.

To further evaluate the reactivity of rabbit antibodies, Western blot analysis was performed using rHlFERs, whole tick homogenates, and a midgut homogenate as protein samples. The results showed that sera from immunized rabbits reacted with rHlFERs and the respective native HlFERs from whole ticks and the midgut (Figure 3). Non-specific bands were seen, particularly on partially-fed midguts, which may be due to the reactivity of secondary antibody to rabbit blood proteins, since the ticks were fed to rabbits. In addition, cross-reactivity was further demonstrated here, as the sera from the rHlFER1-immunized rabbit showed a positive band against rHlFER2, while the sera from the rHlFER2-immunized rabbit showed a positive band against rHlFER1. Both immune sera also reacted with rHlPrx2 but to a lesser extent. No positive bands were detected in any of the tested protein samples using sera from control rabbits.

### Tick infestation challenge

**Figure 4:**
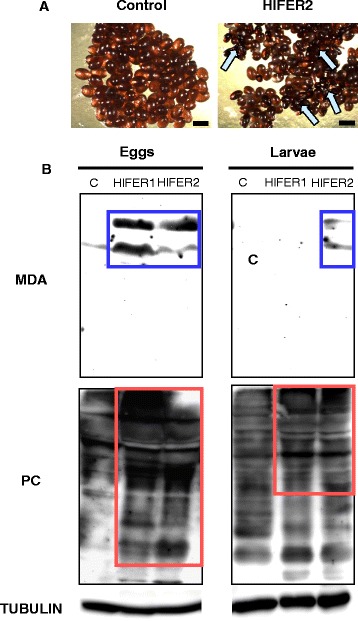
**Effect of recombinant HlFER vaccination on the eggs and larvae. (A)** The morphology of eggs laid by ticks infested on immunized rabbits was compared. Eggs with abnormal morphology, indicated by arrows, were observed from ticks infested on the rHlFER2-vaccinated group but not from the ticks from the control group. Bars = 200 μm. **(B)** Detection of oxidative stress in the eggs and larvae. Malondialdehyde (MDA), a product of lipid peroxidation, and protein carbonyl (PC) resulting from protein oxidation were detected in the eggs and larvae of ticks from vaccinated rabbits using specific immunoblot detection kits. Tubulin was detected as internal control. Proteins with increased MDA and PC are enclosed in blue and red boxes, respectively. C, eggs/larvae from a tick infested on a control rabbit; HlFER1, eggs/larvae from a tick infested on a rHlFER1-vaccinated rabbit; HlFER2, eggs/larvae from a tick infested on a rHlFER2-vaccinated rabbit.

The rabbits were infested with adult *H. longicornis* after two weeks from the third immunization. The total number of attached and engorged ticks from rHlFER1-immunized, rHlFER2-immunized, and control rabbits was 78, 80 and 78, respectively, which was not significantly different. After dropping, the ticks were collected and evaluated for blood feeding and reproduction parameters (Table 1). Ticks infested on rHlFER1- and rHlFER2-immunized rabbits had a lower engorged weight compared to that of the control. However, a significant difference (*P* <0.0001) was only observed in ticks infested on rHlFER2-immunized rabbits, with a 16% mean reduction in engorged weight (R_W_). Moreover, the engorged weight of ticks from rHlFER2-immunized rabbits was also significantly lower (*P* = 0.0377) compared to that of ticks from rHlFER1-immunized rabbits. No mortalities were observed in any of the groups until the completion of egg laying. Eggs with abnormal features, such as irregular shape, wrinkled surface and darker colour, were observed from ticks infested on rHlFER2-immunized rabbits (Figure 4) but not from the ticks infested on control and rHlFER1-immunized rabbits. Upon completing oviposition, the eggs were weighed and the average egg weight for each group was calculated. Ticks from both rHlFER1- and rHlFER2-immunized rabbits had a significantly lower egg weight (*P* <0.05). The ticks from rHlFER2-immunized rabbits had the least egg weight among the three groups, significantly lower (22.4% reduction, *P* <0.0001) compared to the control group, but not compared to the rHlFER1 group. The effect of vaccination on hatching was evaluated by the number of ticks with completely hatched eggs. Both rHlFER groups had a reduced number of ticks with completely hatched eggs (~20% reduction) compared to the control group. Furthermore, larval mortality was observed from some ticks in these groups, but not from control. Based on these parameters, the calculated vaccine efficacy (E) for rHlFER1 is 34% and for rHlFER2 is 49%.

**Table 1:** **Effect of vaccination using recombinant HlFERs on tick feeding and reproduction parameters**

**Experimental group**	**Engorged weight (mg)**	**R**_**W**_^**a**^ **(%)**	**Egg weight (mg)**	**R**_**O**_^**a**^ **(%)**	**Ticks with hatched eggs (%)**	**R**_**H**_^**a**^ **(%)**	**Ticks with dead larvae (%)**	***E*** ^**b**^ **(%)**
**Adjuvant (control)**	261.2 ± 51.9	0	170.0 ± 40.4	0	100.0	0	0	--
**rHlFER1**	243.4 ± 77.3	6.8	142.8 ± 62.5*	12.4	82.0*	18.0	5.5	34.0
**rHlFER2**	218.0 ± 66.0*	16.5	126.5 ± 55.8*	22.4	80.0*	20.0	5.7	49.0

Data represent average values from ticks infested on three rabbits for each group, from two separate vaccination trials.

^a^Formulas for the calculation of reductions in engorged weight (R_W_), oviposition (R_O_), and hatch (R_H_) are described in the Methods section.

^b^Vaccine efficacy (E) was calculated by comparing tick engorged weight, tick egg weight, and the number of ticks with completely hatched eggs from the recombinant HlFER1 (rHlFER1) or recombinant HlFER2 (rHlFER2) group with those from the control group. The formula is described in the Methods section.

*Significantly different vs. control (*P* < 0.05, Student’s *t-*test).

### Effects of antibodies on eggs and larvae

Among the functions of ferritins in ticks is the prevention of iron-mediated oxidative stress [18]. Compared with the previous results of *Hlfer* gene silencing [17], we observed less pronounced effects on the blood feeding, survival, and egg production of adults. Therefore, we analyzed the eggs and larvae to elucidate the mechanism by which antibodies against HlFERs can affect the ticks. We first detected whether the host antibodies against rHlFERs are present in the eggs (Additional file 2: Figure S2). Western blot analysis showed positive bands for rHlFER1 when egg homogenates from ticks infested on rHlFER1- and rHlFER2-immunized rabbits were used as primary antibodies. Meanwhile, a positive band for rHlFER2 was detected when the egg homogenate from ticks infested on rHlFER2-immunized rabbits was used as the primary antibody. No bands were detected when egg homogenates from ticks infested on control rabbit, and secondary antibody alone was used. We then evaluated whether the antibodies can block the function of native HlFER2. Western blot analysis showed that only HlFER2 is present in the eggs and larvae from all groups as we have previously demonstrated [17]. We next detected two oxidative stress biomarkers, MDA from lipid peroxidation and protein carbonyl from protein oxidation [22], using specific antibodies. MDA was detected in the eggs from ticks infested on rHlFER1- and rHlFER2-immunized rabbits and in larvae from ticks infested on rHlFER2-immunized rabbits (Figure 4B). Meanwhile, the amount of protein with the carbonyl group was higher in eggs and larvae from ticks infested on rHlFER1- and rHlFER2-immunized rabbits than from the control group (Figure 4B). These results suggest the occurrence of oxidative stress in these samples.

## Discussion

The use of vaccines for tick and tick-borne pathogen control has many advantages over the application of chemical acaricides in terms of resistance development and environmental and animal product contamination concerns. Thus, numerous tick studies are now focused on developing anti-tick vaccines, but most have limited effectiveness in a wide range of tick-species. An anti-tick vaccine affecting multiple tick species and targeting both the ticks and pathogens is still far from reality [13]. Multiple approaches are currently being employed to screen tick antigens and evaluate their potential [12]. Our previous studies on two ferritins of the hard tick *H. longicornis* using RNAi showed that both are physiologically important in blood feeding and reproduction [17] by preventing iron overload and the occurrence of oxidative stress [18]. Furthermore, a previous study showed that FER2 vaccination had considerable effects on infestation of *I. ricinus* and *R. microplus* [19]. These prompted us to evaluate and compare the potential of two HlFERs as targets for the control of *H. longicornis*.

HlFERs are abundant in different tissues of the tick, with HlFER1 being intracellular in nature, while HlFER2 is secretory [17]. Being concealed antigens, these proteins are not normally encountered by the host immune system during blood feeding, and thus a high level of host antibodies against HlFERs is probably necessary for the blockade of the HlFER function [23]. HlFERs also have complex structures that might require more antibodies binding on them before their function is blocked. The increasing antibody titer after immunization with rHlFERs showed that these proteins are immunogenic. Furthermore, the antibody titer in the immunized rabbits after infestation was as high as the titer after the third immunization, implying that the ticks must have ingested a high amount of anti-rHlFER antibodies during blood feeding. The effects of the antibodies on adult ticks were seen in the reduced engorged bodyweight and oviposition, although these were lower compared to the results of *Hlfer* gene silencing [17], demonstrating differences in the mechanisms by which RNAi and host antibodies exert their blockade effects against a certain molecule. In contrast to the vaccination result against *I. ricinus* and *R. microplus* using FER2 [19], no effect on attachment and number of engorging ticks was observed in either group. Nevertheless, the ability of host antibodies to react with native tick HlFERs in the whole tick and midgut shown in Western blot analysis suggests that the antibodies can bind with native HlFERs within the ticks and possibly interfere with their crucial function on blood feeding and egg production. The cross-reactivity of the antibodies to recombinant proteins seen in ELISA and Western blot analyses may be partly attributed to the His-tag, since the antibodies also reacted to HlPrx2. However, since the amino acid sequences of HlFER1 and HlFER2 have 40% identity, there is still a possibility that the antibodies against tick HlFER1 may cross-react with tick HlFER2, and vice versa.

We wanted to elucidate the mechanism by which antibodies against HlFERs can affect the ticks. It has been demonstrated previously that host antibodies can pass through the midgut barrier of ticks and circulate in the hemolymph [24–26]. Detection of positive bands for recombinant HlFERs on Western blot analysis using egg homogenates as primary antibodies may indicate the presence of antibodies against rHlFERs in the eggs of ticks infested on rHlFER-vaccinated rabbits. However, further examination is needed to confirm this result. We also observed eggs with abnormal morphology from ticks infested on recombinant HlFER2 rabbits, similar to our observation after *Hlfer2* gene silencing [17]. Only HlFER2 that comes from the adult ticks is present in the eggs, which may serve to supply iron and/or protection against iron overload during embryonic development [17]. The tick embryo and larvae are normally challenged with oxidative stress as embryogenesis and aging progresses [27]. Therefore, the function of antioxidants, including HlFER2, which keeps iron from promoting the formation of reactive oxygen species, is crucial. While it is unclear whether these anti-HlFER antibodies in the eggs are in the free form or are already bound to HlFER2, its presence and the higher level of oxidative biomarkers in the eggs of the ticks from rHlFER-vaccinated rabbits, indicating the occurrence of oxidative stress, are highly suggestive of interference in native HlFER function, eventually leading to embryonic death and, hence, reduced hatching. Furthermore, the persistence of these antibodies in the larvae after hatching most likely caused oxidative stress and larval mortality. Thus, there is also a possibility that these antibodies may interfere with HlFERs when the larvae feed on a host.

The results of the tick challenge after vaccination showed that rHlFER2 has higher vaccination efficacy than rHlFER1. Although our previous findings suggested that HlFER1 is the major iron-storage HlFER, abundant in most tick tissues, and that silencing the *Hlfer1* gene seemed to have a greater impact on blood feeding, survival and reproduction [17], the importance of HlFER2 as an iron transporter in hard ticks should not be overlooked. Moreover, the mainly intracellular localization of HlFER1 might have made it inaccessible to anti-HlFER1 antibodies, with the exception of the midgut, where the antibody may interact with HlFER1 within digestive cells. On the other hand, HlFER2, being a secretory protein, may be more accessible to the antibodies after passing through the midgut barrier. HlFER2 is abundant in the hemolymph, circulating within the tick’s body, and as mentioned earlier, can be passed to the eggs. This systemic function of HlFER2, as well as its exclusive presence in the eggs, may have contributed to its higher vaccine efficacy. Furthermore, the antibodies against HlFER2 showed a higher cross-reactivity compared to antibodies against HlFER1.

Ferritin is a highly conserved molecule among different tick species that is ubiquitous in most tick tissues and in all developmental stages. HlFERs have high homology with other tick ferritins. This makes tick ferritins a highly preferable candidate target antigen for the formulation of a multi-species anti-tick vaccine [13]. In contrast to HlFER1, HlFER2 has a lower identity/similarity, less than 40%, to vertebrate ferritins [17]. The secretory FER2 is also considered unique to ticks [16]. In the light of the results in this study, HlFER2 is a better antigen than HlFER1, supporting the previous vaccination study in other tick species [19].

## Conclusion

The importance of ticks as ectoparasites of humans and animals and vectors of several diseases is widely known. Vaccination is highly anticipated to overcome the drawbacks of chemical acaricide control. Thus, numerous tick studies are aiming to identify a single or multiple target antigens that can affect multiple tick species and, ideally, also target tick-borne pathogens [1,12,13]. Most of the tick vaccination studies focus on controlling *Rhipicephalus* and *Ixodes* species. Here we investigated and compared the potential of two HlFERs as targets for the control of the hard tick *H. longicornis*. RNAi is a good technique for screening candidate antigens for vaccine development [12]. Our previous gene silencing studies showed that HlFERs are crucial to the blood feeding and reproduction of *H. longicornis* [17], providing protection against iron-mediated oxidative stress [18] and making them good candidate vaccine antigens. Our vaccination experiments showed that both rHlFER1 and rHlFER2 are highly immunogenic, inducing host antibody production. The tick infestation challenge showed that immunizing the host with rHlFER2 significantly reduced the engorged weight of the infested ticks. Immunization with either of the rHlFERs reduced the number of eggs and the number of ticks with completely hatched eggs, with rHlFER2 producing a greater reduction. Based on these tick parameters, rHlFER2 showed a higher vaccine efficacy of almost 50%. Moreover, eggs with abnormal morphology were observed from ticks infested on rHlFER2-immunized rabbits. We also attempted to elucidate the mechanism of anti-HlFER antibody protection against ticks. Induced antibodies against rHlFERs were detected in the eggs. The presence of a higher level of molecules produced during lipid and protein oxidation in the eggs and larvae from ticks infested on rHlFER-vaccinated rabbits indicates the occurrence of oxidative stress, suggesting that the antibodies interfered with the HlFER2 function. Collectively, our results show that the secretory HlFER2 is a good target for the control of *H. longicornis*, supporting the findings of a previous study targeting FER2 in other hard tick species [19]. While its vaccine efficacy may still be lower compared with those of other studied vaccine antigens, inclusion of HlFER2 in the vaccine with other antigens may yield better results than immunizing with a single kind of antigen and may provide multi-species protection since FER is a highly conserved molecule.

## Additional files

Additional file 1: Figure S1. ELISA using recombinant *H. longicornis* peroxiredoxin (HlPrx2) as antigen. Sera collected on days 0, 7, 21 and 35 from recombinant HlFER1 (rHlFER1), recombinant HlFER2 (rHlFER2) and Control rabbits were checked for reactivity against rHlPrx2. Additional file 2: Figure S2. Detection of antibodies in the eggs of ticks infested on vaccinated rabbits. (A) Induced antibodies from immunized rabbits were detected in the eggs by Western blot analysis using recombinant HlFER1 (rHlFER1) and recombinant HlFER2 (rHlFER2) as protein samples. Egg homogenates from ticks infested on the control (C), rHlFER1- (HlFER1), and rHlFER2- (HlFER2) vaccinated rabbits were used as primary antibodies. Polyclonal HRP-conjugated goat anti-rabbit immunoglobulins were used as secondary antibodies to detect rabbit antibodies. Arrows point to positive bands for rHlFER1 or rHlFER2. (B) As an additional control, the membrane was incubated with polyclonal HRP-conjugated goat anti-rabbit immunoglobulins only.

## Abbreviations

ELISA: Enzyme-linked immunosorbent assay
FER: Ferritin
HlFER1: *Haemaphysalis longicornis* ferritin 1 (intracellular)
HlFER2: *H. longicornis* ferritin2 (secretory)
HRP: Horseradish peroxidise
IPTG: Isopropyl β-D-1-thiogalactopyranoside
MDA: Malondialdehyde
ORF: Open reading frame
PBS: Phosphate-buffered saline
PBS-T: PBS with 0.05% Tween 20
PC: Protein carbonyl
rHlFERs: Recombinant *H. longicornis* ferritins
RNAi: RNA interference
R_W_: Reduction of tick engorged weight
R_O_: Reduction of oviposition
R_H_: Reduction of hatching
SDS-PAGE: Sodium dodecyl sulphate polyacrylamide gel electrophoresis
SFTS: Severe fever with thrombocytopenia syndrome
