# Supplementary figures and images for: Evaluation and comparison of the potential of two ferritins as anti-tick vaccines against Haemaphysalis longicornis
Source: Parasit Vectors. 2014 Oct 12;7:482. doi: 10.1186/s13071-014-0482-x (PMC4197249; doi:10.1186/s13071-014-0482-x)

## Slide 1
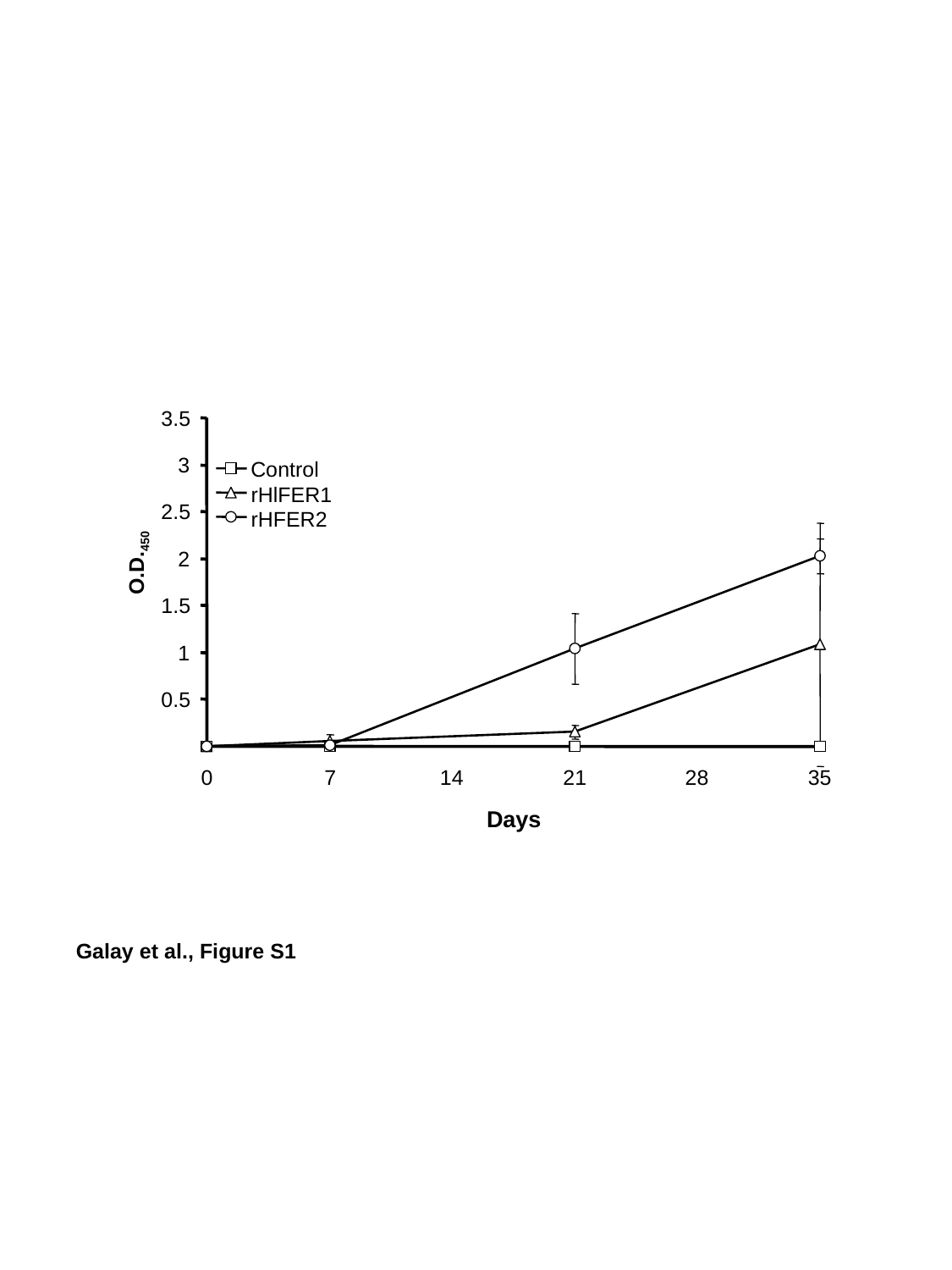

3.5
3
Control
rHlFER1
2.5
rHFER2
2
O.D.450
1.5
1
0.5
0
7
14
21
28
35
Days
Galay et al., Figure S1

Supplement: Additional file 1: Figure S1. — ELISA using recombinant H. longicornis peroxiredoxin (HlPrx2) as antigen. Sera collected on days 0, 7, 21 and 35 from recombinant HlFER1 (rHlFER1), recombinant HlFER2 (rHlFER2) and Control rabbits were checked for reactivity against rHlPrx2. [file 13071_2014_482_MOESM1_ESM.ppt]
