# Supplementary material for: Evaluation and comparison of the potential of two ferritins as anti-tick vaccines against Haemaphysalis longicornis
Source: Parasit Vectors. 2014 Oct 12;7:482. doi: 10.1186/s13071-014-0482-x (PMC4197249; doi:10.1186/s13071-014-0482-x)
Supplement: Additional file 2: Figure S2. — Detection of antibodies in the eggs of ticks infested on vaccinated rabbits. (A) Induced antibodies from immunized rabbits were detected in the eggs by Western blot analysis using recombinant HlFER1 (rHlFER1) and recombinant HlFER2 (rHlFER2) as protein samples. Egg homogenates from ticks infested on the control (C), rHlFER1- (HlFER1), and rHlFER2- (HlFER2) vaccinated rabbits were used as primary antibodies. Polyclonal HRP-conjugated goat anti-rabbit immunoglobulins were used as secondary antibodies to detect rabbit antibodies. Arrows point to positive bands for rHlFER1 or rHlFER2. (B) As an additional control, the membrane was incubated with polyclonal HRP-conjugated goat anti-rabbit immunoglobulins only. [file 13071_2014_482_MOESM2_ESM.ppt]

## Slide 1
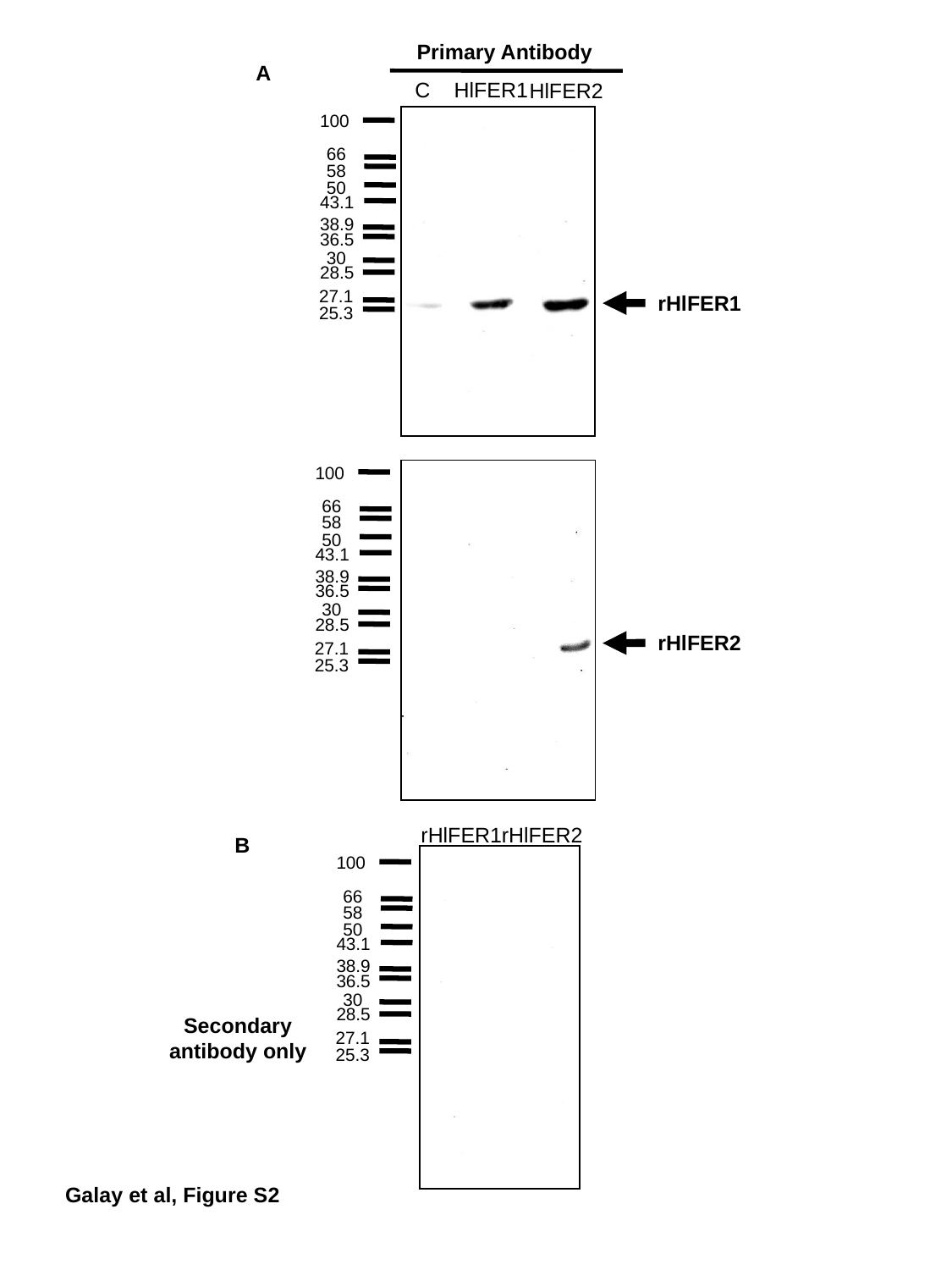

Primary Antibody
A
HlFER1
C
HlFER2
100
66
58
50
43.1
38.9
36.5
30
28.5
27.1
25.3
rHlFER1
100
66
58
50
43.1
38.9
36.5
30
28.5
27.1
25.3
rHlFER2
rHlFER1
rHlFER2
B
100
66
58
50
43.1
38.9
36.5
30
28.5
27.1
25.3
Secondary antibody only
Galay et al, Figure S2
